# Implication of Autophagy in Parkinson's Disease

**DOI:** 10.1155/2013/436481

**Published:** 2013-02-12

**Authors:** Rosa Ana González-Polo, José Manuel Fuentes, Mireia Niso-Santano, Lydia Álvarez-Erviti

**Affiliations:** ^1^Departamento de Bioquímica y Biología Molecular y Genética, E. Enfermería y T.O., Centro de Investigación Biomédica en Red Sobre Enfermedades Neurodegenerativas (CIBERNED), Universidad de Extremadura, 10003 Cáceres, Spain; ^2^INSERM U848, Institut Gustave Roussy, Pavillon de Recherche 1, 94805 Villejuif, France; ^3^Department of Clinical Neuroscience, UCL Institute of Neurology, London NW3 2PF, UK

Autophagy is an intracellular catabolic mechanism mediated by lysosomes, which is responsible for most of the degradation and recycling of cytoplasmic components and intracellular dysfunctional or damaged organelles. Increasing evidences suggest that autophagic deregulation causes accumulation of abnormal proteins or damaged organelles, which is a characteristic of chronic neurodegenerative conditions, such as Parkinson's disease (PD), a multifactorial disorder, which is neuropathologically characterized by age-dependent neurodegeneration of dopaminergic neurons in the midbrain. Indeed, promoting the clearance of aggregate-prone proteins via pharmacological induction of autophagy has proved to be an useful mechanism for protecting cells against the toxic effects of these proteins in the context of neurodegenerative diseases and protecting neurons from apoptosis. This special issue is composed of seven excellent reviews addressing the analysis of different models of Parkinsonism in which autophagy plays a key role.

The paper entitled “*Parkinson's disease and autophagy*” by E. Sánchez-Perez et al. reviews some of the mechanisms underlying gene mutations associated with autophagy in PD familial cases. The authors expose as both deficits and stimulation of autophagy underlie with neurodegeneration, suggesting that altered protein and organelle clearance, either by excess or deficit, are involved in the onset of PD. They claim that the mechanisms that could explain this apparently paradoxical behavior are not clear, and further investigation is required in order to use the autophagy machinery and mitochondria and protein-aggregates removal as an effective and safe therapeutic strategy in the treatment of familial and sporadic PD. 

The paper “*Dopamine oxidation and autophagy*” by P. Muñoz et al. resumes as aminochrome (a dopamine derevative) has been proposed to play an essential role in the degeneration of dopaminergic neurons containing neuromelanin by inducing autophagy dysfunction in these cells. In this sense, aminochrome is able to induce the formation of *α*-synuclein protofibrils that inactivate chaperone-mediated autophagy and also the formation of adducts with *α*- and *β*-tubulin, which induce the aggregation of the microtubules required for the fusion of autophagy vacuoles and lysosomes. The authors conclude that aminochrome is clearly implicated in the dysfunction of protein degradation in dopaminergic neurons.

In the paper entitled “*Mitochondrial dynamics and mitophagy in the 6-hydroxydopamine preclinical model of Parkinson's disease*”, M. F. Galindo et al. discuss the participation of mitochondrial dynamics and autophagy in the 6-hydroxidopamine-induced PD model. They focus your attention on the regulation of dynamic mitochondrial processes such as fusion, fission, and mitophagy, with special emphasis in the role of the second messengers and reactive oxygen species as well as mitochondria as the headquarters of cell death. Finally this paper highlights the therapeutic potential of small-molecule inhibitors of mitochondrial division in PD. 

The article “*Methamphetamine and Parkinson's disease*” by N. Granado et al. focuses the role of methamphetamine in PD. This review shows that methamphetamine, an amphetamine-type stimulant which actually is the second most widely used illicit drug in the world, damages dopaminergic neurons in the substantia nigra, resulting in a significant loss of dopamine in the striatum. Biochemical and neuroimaging studies evidence that molecular changes are similar to those observed in PD patients.

In relation with the previous review, the paper “*N-Acetyl cysteine protects against methamphetamine-induced dopaminergic neurodegeneration via modulation of redox status and autophagy in dopaminergic cells*” by P. C. Shivalingappa et al. reveals that the loss of cellular levels of glutathione is one of the pivotal mechanisms involved in methamphetamine-induced neurotoxicity and autophagy in mesencephalic dopaminergic neuronal cells. They claim that the treatment with N-acetyl cysteine partially reverses methamphetamine-induced apoptotic cell death, possibly by replenishing glutathione levels. Interestingly this paper is the first report demonstrating that N-acetyl cysteine pretreatment can ameliorate methamphetamine-induced autophagy, highlighting the importance of redox status of the cell in methamphetamine-induced dopaminergic neurodegeneration.

J. M. Bravo-San Pedro et al. discuss in the review article entitled “*Parkinson's disease: leucine-rich repeat kinase 2 and autophagy, intimate enemies*” the role of leucine-rich repeat kinase 2 in autophagy and how the deregulations of this degradative mechanism in cells can be implicated in the PD etiology. This review claims that LRRK2 protein is involved in cellular autophagy through direct modulation, the alteration of its own kinase activity, or the mediation of autophagy in response to external stimuli. Therefore, the authors affirm that is important to understand the activity of LRRK2 to elucidate the cellular death that has been identified in studies of *park8* mutations. In this paper the authors conclude that this knowledge would be essential for the development of strategies for reducing the cellular sensitivity and cell death that could trigger the development of PD.

Finally, P. Gómez-Suaga et al. delve into the relationship between autophagy and LRRK2 in the review article entitled “*A link between autophagy and the pathophysiology of LRRK2 in Parkinson's disease*” where they discuss current knowledge about mechanism(s) by which mutant LRRK2 may regulate autophagy, which highlights additional putative therapeutic targets. They claim that currently many questions remain to be addressed, such as whether TPCs (or NAADP binding proteins) are LRRK2 targets, whether LRRK2 causes indeed measurable changes in intracellular calcium levels, or how LRRK2 regulates the activity or localization of distinct rab proteins. In this sense, authors stated that further work would be needed for delineating the precise molecular links between LRRK2, autophagy, and NAADP-mediated events.

The compilation of these reviews included in this special issue of the relationship between PD, and autophagy, shows that there is much controversy in this field. Currently there are many research lines that require a lot of work to get there someday to be able to clarify a possible route of finding treatments against PD based on the mechanism of autophagy.

*Rosa Ana González-Polo* *Rosa Ana González-Polo* *José Manuel Fuentes* *José Manuel Fuentes* *Mireia Niso-Santano* *Mireia Niso-Santano* *Lydia Álvarez-Erviti* *Lydia Álvarez-Erviti*

